# Isolation and Characterization of Group III *Campylobacter jejuni*–Specific Bacteriophages From Germany and Their Suitability for Use in Food Production

**DOI:** 10.3389/fmicb.2021.761223

**Published:** 2021-12-09

**Authors:** Severin Michael Steffan, Golshan Shakeri, Jens Andre Hammerl, Corinna Kehrenberg, Elisa Peh, Manfred Rohde, Claudia Jackel, Madeleine Plotz, Sophie Kittler

**Affiliations:** ^1^Institute for Food Quality and Food Safety, Foundation University of Veterinary Medicine Hannover, Hanover, Germany; ^2^Department of Food Hygiene and Aquaculture, Faculty of Veterinary Medicine, Ferdowsi University of Mashhad, Mashhad, Iran; ^3^Department Biological Safety, German Federal Institute for Risk Assessment, Berlin, Germany; ^4^Institute for Veterinary Food Science, Justus-Liebig-University Giessen, Giessen, Germany; ^5^Central Facility for Microscopy, Helmholtz Centre for Infection Research GmbH, Braunschweig, Germany

**Keywords:** *Campylobacter virus*, phage biocontrol, one health approach, phage therapy, novel antimicrobials, poultry meat, efficiency of plaquing, bacteriophages

## Abstract

*Campylobacter* spp. are a major cause of bacterial foodborne diarrhea worldwide. While thermophilic *Campylobacter* species asymptomatically colonize the intestines of chickens, most human infections in industrial countries have been attributed to consumption of chicken meat or cross-contaminated products. Bacteriophages (phages) are natural predators of bacteria and their use at different stages of the food production chain has been shown to reduce the public health burden of human campylobacteriosis. However, regarding regulatory issues, the use of lytic phages in food is still under discussion and evaluation. This study aims to identify lytic phages suitable for reducing *Campylobacter* bacteria along the food production chain. Therefore, four of 19 recently recovered phages were further characterized in detail for their lytic efficacy against different *Campylobacter* field strains and their suitability under food production settings at different temperatures and pH values. Based on the results of this study, the phages vB_CjM-LmqsCP1-4 and vB_CjM-LmqsCP1-5 appear to be promising candidates for the reduction of *Campylobacter jejuni* in food production settings.

## Introduction

Campylobacteriosis is the most frequently reported foodborne bacterial gastroenteritis in the European Union (EU) since 2005 (European Food Safety Authority [EFSA] and European Centre for Disease Prevention and Control [ECDC], 2021), but represents also an important zoonosis worldwide (Skarp et al., 2016). In 2019, the European Food Safety Authority (EFSA) notified 220,682 campylobacteriosis cases in the EU. While 49.8% of the reported cases were acquired in the EU, 3% were considered to originate from other countries. However, for 47.2% of the human cases, further information on the travel status or the country of infection was unknown (European Food Safety Authority [EFSA] and European Centre for Disease Prevention and Control [ECDC], 2021). In general, consumption of broiler meat is considered to be the most common cause for human infections (European Food Safety Authority [EFSA], 2010). Thermophilic *Campylobacter* spp. have been shown to be the most prevalent cause of campylobacteriosis. *Campylobacter (C.) jejuni* and *C. coli* are the most frequent species, while *C. lari, C. fetus* and *C. upsaliensis* have been detected less frequently in campylobacteriosis cases (European Food Safety Authority [EFSA] and European Centre for Disease Prevention and Control [ECDC], 2021). Symptoms of *Campylobacter* enteritis include watery to hemorrhagic diarrhea and abdominal pain. Severe long-term sequelae like the Guillain-Barré syndrome, reactive arthritis, and erythema nodosum can occur in rare cases subsequent to the infection (Nachamkin, 2002; Moore et al., 2005; Skarp et al., 2016).

*Campylobacter jejuni* and *C*. *coli* can colonize the intestine of various wild, domestic, and farm animals (Mulder et al., 2020; European Food Safety Authority [EFSA] and European Centre for Disease Prevention and Control [ECDC], 2021). In broiler flocks, they can usually be detected after 2–3 weeks of housing and spread rapidly until almost all animals of the flock are colonized without showing clinical symptoms (Shreeve et al., 2000; Newell and Fearnley, 2003; Newell et al., 2011). During defeathering, evisceration, and further processing, the pathogens can be transferred to the carcasses (Lee and Newell, 2006; Sasaki et al., 2013), and by cross-contamination during food preparation, contamination of other foodstuffs can occur (Luber et al., 2006; Cardoso et al., 2021). Current estimations predict that a reduction in the *Campylobacter* load in chicken ceca by 2 log_10_ units can diminish the risk for human infection, arising from consumed broiler meat, by 42%. However, a 3 log_10_ unit reduction would lead to a 58% lower risk for human infections (European Food Safety Authority [EFSA] et al., 2020; European Food Safety Authority [EFSA] and European Centre for Disease Prevention and Control [ECDC], 2021).

Bacteriophages (phages) are viruses that specifically infect bacterial genera, species or strains. Currently, the International Committee on Taxonomy of Viruses (ICTV) recognizes nine *Campylobacter* viruses (ICTV, 2019) that all have an AT-rich double stranded DNA and belong to the family *Myoviridae* (Bradley’s morphotype A1) (Sails et al., 1998) in the order of *Caudovirales*. Phages in this family are non-enveloped viruses, with a head-tail structure encompassing a contractile tail. *Campylobacter* viruses which are part of the *Siphoviridae* family, have recently been described, but are not yet recognized by the ICTV (Nowaczek et al., 2019). The ICTV currently recognizes a scheme based on sequence analysis that subdivides the family into the two genera Firehammerviruses, including four recognized members [type species CP220, formerly named Cp220likevirus (2014) and Cp220virus (2015–2018)], and Fletcherviruses, including five recognized members [type species CP8, formerly named Cp8unalikevirus (2014) and Cp8virus (2015–2018)] (Javed et al., 2014; Adams et al., 2016; ICTV, 2019; Jackel et al., 2019). Moreover, there are currently two unclassified Firehammerviruses, as well as one classified and 21 unclassified Fletcherviruses listed by the NCBI taxonomy browser (Schoch et al., 2020). A former typing scheme divides *Campylobacter* phages into three groups based on their genome sizes (group I ∼320 kb, group II ∼184 kb, and group III ∼138 kb). These groups could also be predicted according to their prevailing host specificity (Sails et al., 1998; Javed et al., 2014). Fletcherviruses were assigned to phage group III and Firehammerviruses to phage group II before ICTV classification was established (Hammerl et al., 2011; Javed et al., 2014; Jackel et al., 2019; Ushanov et al., 2020).

The restricted host range of the *Campylobacter* phages described so far allows specific targeting of pathogenic species (Richards et al., 2019), but at the same time raises the need to combine various phages in cocktails for effective applications in food production settings, where different, unknown *Campylobacter* strains might be encountered (Chan et al., 2013; Kittler et al., 2013). Combining phages also offers the advantage of preventing bacterial resistance development that may occur as a result of phage application (Fischer et al., 2013; Azam and Tanji, 2019). Nonetheless, while some phage-resistant bacterial cells might occur even if cocktails were used (Fischer et al., 2013), phage-resistant bacteria were shown to exhibit a reduced colonization fitness (Kittler et al., 2014; Oechslin, 2018; Azam and Tanji, 2019). Phages can be used at multiple stages of the food production process without changing the organoleptic properties of the products, such as odor or taste. They can be applied during meat production pre- or post-harvest, and for bio sanitation (Connerton et al., 2011; Polaska and Sokolowska, 2019) as mandated by a farm to fork strategy (Nauta et al., 2007). However, a lack of data and approval procedures still impedes the implementation of phages as an efficient alternative for *Campylobacter* biocontrol (European Food Safety Authority [EFSA], 2009; European Food Safety Authority [EFSA] et al., 2020).

Application of *Campylobacter* phages in food production settings has been considered as a promising technology (European Food Safety Authority [EFSA] et al., 2020; Zbikowska et al., 2020). Previous studies highlighted the need for (i) a lytic phage collection directed against current *Campyobacter* field strains (Carrillo et al., 2005), (ii) more precise and faster methods to identify and select useful phages (Xie et al., 2018; Zbikowska et al., 2020), and (iii) a better understanding of bacteriophage kinetics to allow for advanced considerations concerning dosing and timing of phage applications (Cairns et al., 2009; Hammerl et al., 2014; Loessner et al., 2020). Furthermore, genome determination of *Campylobacter* phages was also shown to be laborious due to prevailing DNA modification and extensive repetitive sequences, including hypermutable polyGtracts, resulting in low amounts of extracted DNA and/or incomplete genomes (Hammerl et al., 2011; Carvalho et al., 2012; Jackel et al., 2015; Crippen et al., 2019; Sorensen et al., 2021). However, all *Campylobacter* phages described to date are closely related in their genetic composition and exhibit highly conserved nucleotide sequences. To the best of our knowledge, all *Campylobacter* phages are free of genes encoding antimicrobial and/or virulence factors (Hammerl et al., 2015). Questions regarding the stability of the phage genomes are hard to evaluate as *Campylobacter* phages comprise homing endonucleases, which might be involved in adaption of the genomes according to the prevailing selection pressures (i.e., host specificity etc.). This study represents an initiative to increase the number of carefully characterized phages against *C. jejuni*. Furthermore, we aimed to determine differences in the performance of phages with similar host ranges or origins in liquid cultures of *Campylobacter* field strains and analyzed their stability upon storage, varying pH values and temperatures (Carrillo et al., 2005; Jonczyk et al., 2011) for estimating their suitability for practical application under real life settings.

## Materials and Methods

### Bacteria Strains, Typing and Growth Conditions

Information on the bacterial strains used in this study are summarized in Supplementary Table 1. Briefly, the used bacterial panel consisted of two type strains (DSM 4688, DSM 4689) and two reference strains (NCTC 11168, ATCC BAA-2151) as well as 23 field isolates from chicken samples collected from commercial poultry farms in Lower Saxony, Germany, in 2017 (Supplementary Table 1). Field isolates were typed to preselect a host panel representing a wide range of current field isolates by *flaA*-typing in accordance with Zhang et al. (2018) followed by analysis of *flaA* sequences (Eurofins NDSC Food Testing GmbH, Hamburg, Germany). SmaI-PFGE macrorestriction analysis of the bacterial isolates was conducted as previously described (Nawaz et al., 2003) with minor modifications, using a CHEFIII System (BioRAD laboratories GmbH, Feldkircen, Germany).

*Campylobacter* spp. stock cultures were stored at –80°C. Bacteria were cultivated on Columbia Agar sheep Blood “Plus” plates (Thermo Fischer Scientific Oxoid Deutschland GmbH, Wesel, Germany) at 42°C under microaerobic conditions (5% O_2_, 10% CO_2_, 85 N_2_, >80% humidity). Liquid cultures were prepared with brain-heart infusion (Carl Roth GmbH & Co., KG, Karlsruhe, Germany, X916) supplemented with 1 mM calcium chloride (CBHI).

### Bacteriophage Isolation and Propagation

The soft-agar overlay technique was used to detect *Campylobacter* phages from different matrices. All samples (n_cecal_ = 136, n_fecal_ = 111, n_neck skin_ = 54) originated from poultry farms in Lower Saxony, Germany. For sample preparation, approximately 2 g of fecal or cecal samples were dispersed in 10 ml SM-buffer [100 mM NaCl, 8 mM MgSO_4_, 50 mM Tris-HCl (pH 7.5)] using an Ultra-Turrax T10 homogenizer (IKA-Werke GmbH & Co. KG, Staufen, Germany). In contrast, skin samples were thoroughly rinsed by massaging them in a plastic bag containing 10 ml SM-buffer. After overnight shaking at 4°C, the samples were centrifuged twice (1st step at 3,488 × g for 20 min, 2nd step at 13,000 × g for 10 min), and the supernatant was filtered through a 0.2 μm polyethylensulfon membrane (PES) syringe filter (Carl Roth GmbH & Co., KG). For further purification, the resulting filtrate was cocultured with *C. jejuni* strain NCTC 12662 as previously described (Fischer et al., 2013) with minor modifications. Briefly, bacterial overnight cultures were cultivated on blood agar plates for 14–15 h, and a suspension in 10 mM magnesium sulfate was adjusted to a McFarland of 1.0 (McF) (DEN-1 densitometer, Grant Instruments Grant Bio, Thermo Fischer Scientific GmbH, Schwerte, Germany). One-milliliter of bacterial suspension was incubated in 9 ml culture medium for approximately 4 h. Afterward, 100 μl of the culture was mixed with the sample filtrate, and incubated for a further 10 min. Subsequently, the mixture was added to 5 ml molten 0.4% NZCYM-soft agar (Carl Roth GmbH & Co., KG). The soft-agar was poured onto NZCYM plates containing 1.5% agar. After 24 h incubation period, the resulting plaques were purified by a successive threefold picking and plating procedure of single plaques. Subsequent propagation of phages was carried out with 0.7% NZCYM-soft agar until suspensions reached a concentration of approximately 10^8^ plaque forming units (PFU) per ml for host range determination. Concentrations were determined by using serial dilutions of the phage lysates and duplicate plating of 100 μl of each dilution on *C. jejuni* NCTC 12662.

### Host Range Determination and Initial Characterization

The host range of the individual phages was determined in accordance with the plaque assay method of Korf et al. (2020), with some modifications, while the susceptibility of the bacteria was indicated by the efficiency of plaquing (EOP), as defined by Sorensen et al. (2012). In short, square NZCYM-agar plates were overlaid with 7.9 ml NZCYM-soft agar, which had been inoculated with a 2 h pre-incubated liquid culture of one of the 29 *Campylobacter* in CBHI (Supplementary Table 1), and 10 μl of 10-fold serial diluted phage suspensions from each of the 19 phages was applied onto the overlay. Plates were incubated for 20 ± 2 h. Phage/bacteria combinations that produced visible plaques 2–3 times were used to calculate the relative difference of plaque forming ability by dividing the measured concentration on the respective *Campylobacter* isolate or strain by the concentration measured on the host strain NCTC 12662. Production of visible plaques and opaque inhibition zones without plaques in only one of three tests was counted as a negative result.

Initial characterization by HhaI- and VspI***-***macrorestriction with subsequent pulsed-field gel electrophoresis (PFGE) was performed as previously described (Hansen et al., 2007).

### Efficacy Testing of Phages Bacteria Reduction Capability in Liquid Culture

The growth of two *C. jejuni* field strains, Cj18 or LH86, with and without exposure to four different *Campylobacter* bacteriophages vB_CjM-LmqsCP1-4 (CP1-4), vB_CjM-LmqsCP1-5 (CP1-5), vB_CjM-LmqsCP74-2c1 (CP74-2c1), and vB_CjM-LmqsCP132-3c (CP132-3c) at five different multiplicities of infection (MOI_input_ of 10, 1, 0.1, 0.01, and 0.001) (Danis-Wlodarczyk et al., 2021) was examined in liquid cultures using a Tecan Spark Microplate Reader with O_2_ and CO_2_ control, similar to methods described by Xie et al. (2018); Rajnovic et al. (2019), and Zachary et al. (2020) with some modifications. Briefly, phage suspensions were adjusted to 10^8^, 10^7^, 10^6^, 10^5^, and 10^4^ PFU/ml, which were further diluted 10-fold with CBHI to achieve final dilutions of 10^7^, 10^6^, 10^5^, 10^4^, and 10^3^ PFU/ml. Bacterial overnight cultures were adjusted to 3.0 McF in 10 mM MgSO_4_ and used to inoculate 50 ml CBHI that was then incubated for 3 h with shaking (130 rpm). Afterward, the culture was adjusted to 0.5 McF, diluted 100-fold with CBHI and the wells of a 48-well microplate were filled with 250 μl of this suspension and 250 μl of phage suspensions. Plates were incubated with double orbital shaking at 42 °C under microaerobic conditions (5% O_2_, 10% CO_2_, 85% N_2_, 108 rpm). Optical density (OD_600_) was measured hourly for 26 h. There were two replication wells per plate and the experiment was performed in triplicate.

At the end of the experiment, 100 μl were taken from each well with an MOI_*input*_ 10 and 0.001, and plated on mCCDA Agar (Thermo Fischer Scientific Oxoid GmbH, Wesel). After 24 h, one colony per plate was picked and tested for phage resistance by spotting 10 μl of a 10-fold dilution series of phages on NZCYM-soft agar overlays containing the respective bacterial isolate. Bacteria were considered phage-resistant, if no plaques were observed. The remaining culture in the well was treated with 2–3 drops of chloroform and the total phage titer was determined as described above.

### Stability Testing of Phages

Temperature stability was tested by incubating 300 μl of phage suspensions at a concentration of 10^8^ PFU/ml for 15 or 60 min at –20 in a freezer and at 50, 60, 70, or 80°C in a block heater (Grant Instruments Ltd., Royston, United Kingdom). Samples were taken after 0, 15 and 60 min. Experiments were performed in triplicate.

For pH-stability tests, 100 μl of phage suspensions were diluted 10-fold with pH-adjusted phosphate buffer at pH values of 2–12 (Carl Roth GmbH & Co., KG) or tap water to reach a total volume of 1 ml. Suspensions were stored at two temperatures (in an incubator at 22.3 ± 0.4°C or at 41.7 ± 0.7°C) for 24 h. The concentration of each suspension was determined after 24 h. Additional samples were taken for pH 2, 3, and 7 at the start of the experiment (0 h), after 15 min for pH 2, and 120 min for pH 3. Experiments were performed in triplicate.

Stability of phages during storage was assessed by storing 6 ml of phage suspension for 7 months at 4.5 ± 0.5°C or 6 weeks at 23.5 ± 0.7°C. Phage concentrations were determined at regular intervals. The experiment was repeated four times.

### Negative-Staining of Phages

Phages were negatively stained with 2% aqueous uranyl acetate after being adsorbed for 15–30 s onto carbon film according to Valentine et al. (1968). After washing with TE buffer (10 mM TRIS, 1 mM EDTA, pH 6.9) samples were blotted onto filter paper and air dried. Samples were examined in a Zeiss TEM 910 transmission electron microscope (Zeiss, Oberkochen) at an acceleration voltage of 80 kV and at calibrated magnifications with a line replica. Images were recorded digitally with a Slow-Scan CCD-Camera (ProScan, 1,024 × 1,024, Proscan Elektronische Systeme GmbH, Scheuring, Germany) with ITEM-Software (Olympus Soft Imaging Solutions GmbH, Münster, Germany).

### Extraction of Phage DNA and Genome Analysis

Phage suspensions were prepared as described above and phage particles pelleted by centrifugation at 24,000 × g (Avanti J-26S XP, Beckmann Coulter Inc., Brea, CA, United States) for 2 h. The pellet was resuspended with a small amount of SM-buffer and purified by CsCl-gradient (Optima XPN-100, Beckmann Coulter Inc., Brea, United States, 165,100 × g, 4°C, 2 h). Cesium chloride was removed from the phages via dialysis with SM-buffer. The resulting phage suspensions were used for electron micrographs, PFGE macrorestriction analysis and DNA isolation. Phage DNA was extracted from virions purified by cesium chloride density gradient (Sambrook and Russell, 2001) with the Wizard DNA Clean-Up System (Promega, Madison, WI, United States) in accordance with the manufacturer’s instructions, followed by ethanol precipitation.

Phage DNA was subjected to short read whole-genome sequencing in-house at the German Federal Institute for Risk Assessment, Berlin, Germany (BfR). DNA-sequencing libraries were generated using the Nextera XT DNA Library Flex Preparation Kit (Illumina Inc., San Diego, CA, United States) in accordance with the recommendations of the manufacturer. Short read, paired-end sequencing was conducted on different Illumina devices (i.e., MiSeq, NextSeq) using the MiSeq Reagent v3 600-cycle Kit (Illumina). The generated raw reads were subjected to the Aquamis *in house* pipeline (Deneke et al., 2021).

Real-time PCR in accordance with a protocol by Jackel et al. (2017) was performed for phage differentiation. PCR primers targeted the tail tube gene, ORF186 of CP21 for group II phages (CPGII-probe: FAM-CCGGATTGACTGTAGAAACA-BHQ-1) and the gene for the base plate wedge, ORF008 of CP81, for group III phages (CPGII-probe: Cy5-TGTAACTGCCCTGTTTGCTG-BBQ-650).

### Data Analysis

Data preparation, visualization, and statistical analysis (one tailed Student’s *t*-tests and Dunnett’s test) were performed using R software version 4.0.2 (R Core Team, 2020) and the DescTools package version 0.99.40. The ComplexHeatmap package (Gu et al., 2016) was used for visualization of the host range. Phage particle size parameters (head length, head diameter, tail length) were determined by size analysis of virions visible on negatively stained electron micrographs with ImageJ version 1.51q in the Fiji bundle in combination with the ObjectJ plugin. Data analysis of *flaA* typing was performed by PubMLST (Jolley et al., 2018) and SmaI-PFGE macrorestriction interpretation was conducted by GelAnalyzer 19.1 (www.gelanalyzer.com, Istvan Lazar Jr., Ph.D. and Istvan Lazar Sr., Ph.D., CSc.).

## Results

### Phage Isolation and Host Range Determination

In total, 301 samples from chicken sources (chicken feces *n* = 111, cecal content *n* = 136 and neck skin *n* = 54) were examined for the presence of bacteriophages using the soft-agar overlay technique with *C. jejuni* strain NCTC 12662. After threefold serial purification and propagation of plaques, 19 purified phages remained. Four phages were isolated from one fecal sample in 2015, while nine were isolated from cecal samples, and six from chicken neck skin samples in 2017. Information on recovered phages and sample origin are shown in Supplementary Table 2.

**FIGURE 1:**
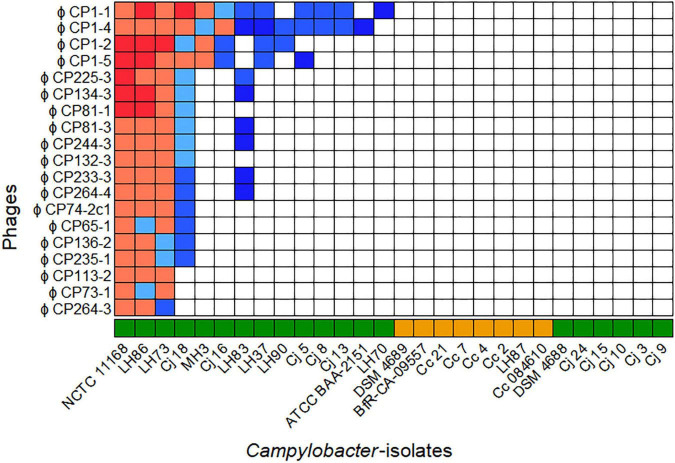
Host ranges of 19 phages isolated in 2015 and 2017 on 28 *Campylobacter* spp. Overall, seven host range patterns could be distinguished. The bacteriophages vB_CjM-LmqsCP1-4 (CP1-4), vB_CjM-LmqsCP1-5 (CP1-5), vB_CjM-LmqsCP74-2c1 (CP74-2c1), and vB_CjM-LmqsCP132-3c (CP132-3c) were selected for further investigations, showing three different host range patterns. Host range evaluation was performed by spotting serial dilutions of bacteriophages onto NZCYM-soft agar overlays inoculated with *Campylobacter* (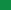 *C*. *jejuni*, 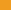 *C*. *coli*). Experiments were performed in triplicate. The concentration (plaque forming units/ml) on different hosts was calculated and divided by the concentration determined on *C*. *jejuni* NCTC 12662. Color coding was used to visualize the EOP values(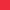
*x* > 1, 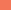
1 ≤ *x* < 0.9, 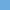 0.9 ≤ *x* < 0.8, 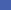 0.8 ≤ *x* < 0.6, 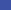 0.6 ≤ *x* < 0, □*x* = 0).

The host ranges of all purified phages were analyzed on 28 different *Campylobacter* isolates (20 *C. jejuni* and 8 *C. coli*) as displayed in Figure 1. All bacterial isolates included showed different *flaA* types (Supplementary Table 1) and/or could be distinguished by SmaI-PFGE macrorestriction analysis (personal communication).

Of the 28 *Campylobacter* isolates tested, 14 *C. jejuni* isolates were susceptible to at least one of the phages, while no plaque formation was observed on six *C. jejuni* and all *C. coli* isolates. The isolated phages displayed seven different lytic profiles. While four phages showed unique patterns, two groups of six and one group of three phages exhibited similar host ranges.

Four bacteriophages showing three of the seven lytic profiles and deriving from three isolation sources were selected for further characterization. The phages CP1-4 and CP1-5 were chosen according to their different host ranges, but they had the same sample origin, while the phages CP74-2c1 and CP132-3c had the same host range, but originated from different samples.

**FIGURE 2:**
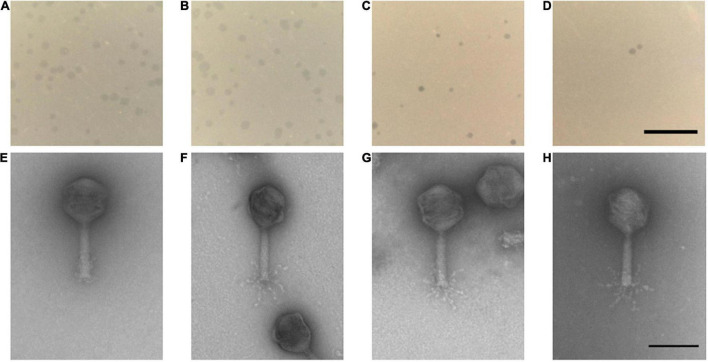
**(A–D)** Plaque morphology of the four examined myovirus phages **(E–H)** and micrographs showing the morphology of the virions. All four phages vB_CjM-LmqsCP132-3c **(A,E)**, vB_CjM-LmqsCP1-5 **(B,F)**, vB_CjM-LmqsCP74-2c1 **(C,G)**, vB_CjM-LmqsCP1-4 **(D,H)** formed clear plaques on *C. jejuni* strain NCTC 12662; scale bar represents 2 mm. The virions consisted of an icosahedral head and a contractile tail structure with tail fibers; negatively stained phage particles with 2% uranylacetate, scale bar represents 100 nm.

The four phages formed clear plaques on their host strain NCTC 12662 as shown in Figures 2A–D. Plaque diameters ranged from 0.75 to 1.86 mm (*n* = 30) after 24 h incubation on 0.7% NZCYM-soft agar (Supplementary Table 3). Negatively stained electron micrographs of all four phages showed that that they had isometric heads, contractile tails and tail fibers, leading to the conclusion that they belonged to the *Myoviridae* family (Figures 2E–H). The mean values for head length, head diameter, and tail length of the phages were very similar, except for CP74-2c1, which displayed a longer tail, and CP1-5, that showed an overall smaller head size than all other analyzed phages (Supplementary Table 3).

### Bacterial Reduction by Phages in Liquid Culture

Growth experiments were performed by measuring optical density (OD_600_) of *C. jejuni* field strains Cj18 and LH86 in liquid cultures with and without exposure to the four phages CP1-4, CP1-5, CP74-2c1, and CP132-3c at five different MOI_input_ (10, 1, 0.1, 0.01, and 0.001), using a Tecan Spark Multiplate reader over a time period of 26 h.

Both bacterial isolates were susceptible to all four phages according to their host ranges (Figure 1), and were chosen for testing the phage’s efficiency for reducing bacterial population growth and overall cell density. The EOP values of the four phages on the two *Campylobacter* isolates varied between 0.93 and 1.07, except for the combinations of Cj18 with CP74-2c1 or CP132-3c: Their EOP values were determined to be approximately 0.8.

**FIGURE 3:**
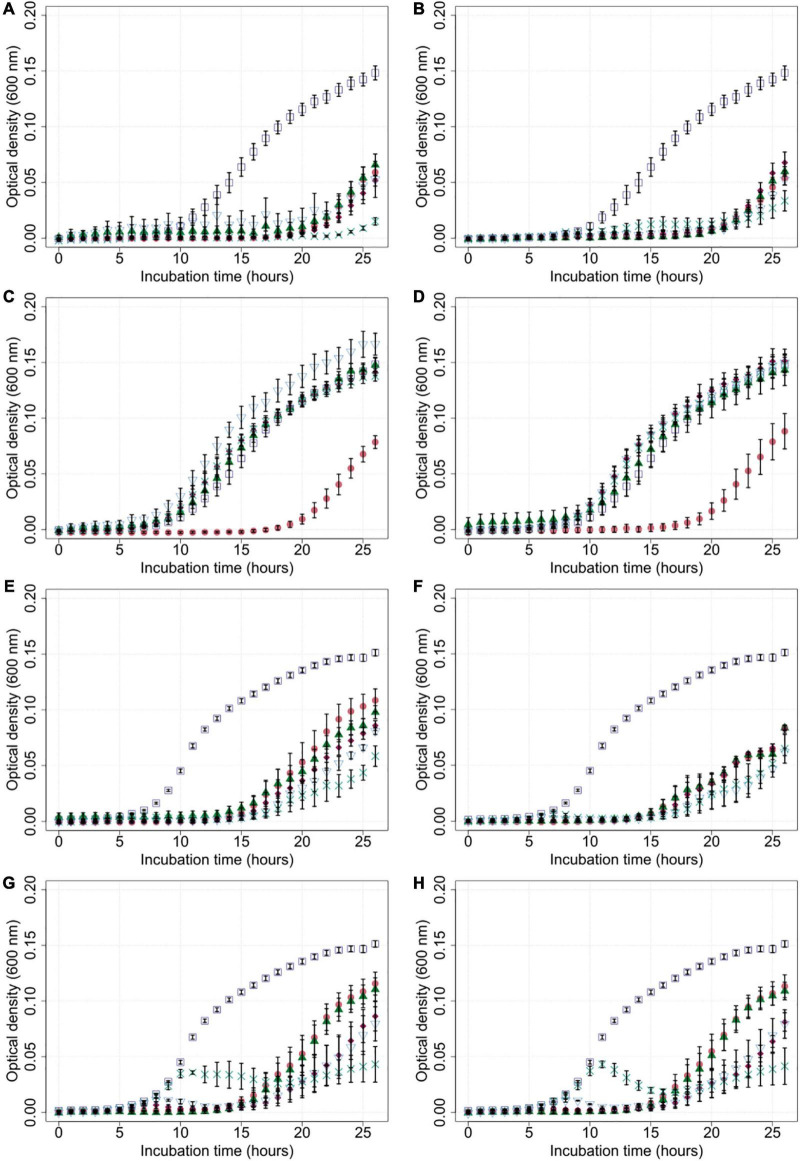
Growth of *Campylobacter* field isolates Cj18 and LH86 in presence of different phages. The growth of *C. jejuni* field strains Cj18 **(A–D)** and LH86 **(E–H)** was inhibited by all phages. Different multiplicities of infection (MOI_input_) used, resulted in different reduction efficiencies. Experiments were performed in a Tecan Spark Microplate Reader under microaerobic conditions at 42°C. Optical density was measured every hour at 600 nm for 26 h. **(A,E)** vB_CjM-LmqsCP1-4, **(B,F)** vB_CjM-LmqsCP1-5, **(C,G)** vB_CjM-LmqsCP74-2c1 and **(D,H)** vB_CjM-LmqsCP132-3c were added to achieve different MOI_input_: 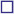 no phages added, 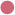 MOI_input_ 10, 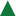 MOI_input_ 1, 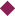 MOI_input_ 0.1, 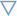 MOI_input_ 0.01, and 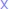 MOI_input_ 0.001. Curves represent mean OD_600_ values of triplicate experiments with two replications per plate. Error bars indicate standard error of the mean.

Generally, bacterial growth was reduced in most liquid cultures that were exposed to bacteriophages compared to the untreated controls (Figure 3). Cultures containing Cj18 and CP74-2c1 or CP132-3c only showed reduced bacterial population growth if a high MOI_input_ of 10 was used in cultures containing the phages. Interestingly, in other experiments using an MOI_input_ of 10, most bacteria grew better than in experiments using a lower MOI_input_ of 0.001 after approx. 20 h (Figures 3C,D). Furthermore the data showed an initial rise and subsequent rapid decrease in optical density for LH86 in combination with CP74-2c1 or CP132-3c at an MOI_input_ of 0.01 and 0.001 (Figures 3G,H).

**FIGURE 4:**
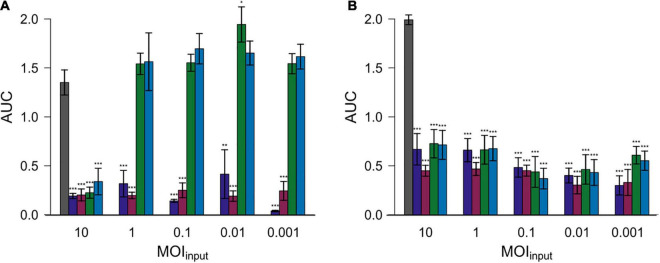
Areas under the curve (AUC) after 26 h for two different *Campylobacter* isolates. *Campylobacter jejuni* Cj18 **(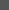 A)** and LH86 **(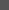 B)** were exposed to four different phages (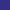 vB_CjM-LmqsCP1-4, 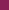 vB_CjM-LmqsCP1-5, 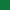 vB_CjM-LmqsCP74-2c1 and 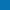 vB_CjM-LmqsCP132-3c) at five different MOI_input_ (10, 1, 0.1, 0.01, and 0.001). The change of optical density over time was recorded. The AUC values were calculated by spline fitting (*t* = 0–26 h). Dunnett’s test with a 95% confidence level was used to compare the AUC values of the exposed samples to the untreated comparisons (significance codes indicate range of *p*-values: *0.01, **0.001, ***>0.001).

Subsequently, the area under the curve (AUC) was calculated by spline fitting of the growth curves (Figure 4). The AUC values for most experiments with added phages were significantly reduced compared to the controls without phage exposure. Nevertheless, the AUC values equaled or exceeded those of experiments without phage exposure in case of experiments in which Cj18 was combined with CP74-2c1 or CP132-3c at an MOI_input_ of 1–0.001.

After each experiment with an MOI_input_ of 0.001 or 10, bacterial isolates were recovered and tested for phage resistance with all four phages. Additionally, final phage concentrations in these wells were determined.

While these tests included only a limited number of bacterial isolates, *Campylobacter* isolates that showed resistance against at least one bacteriophage could be recovered from all experiments (Supplementary Table 4). Interestingly, one of the 27 recovered bacterial isolates was still susceptible to three bacteriophages after the experiment, while seven were susceptible to two phages under the same conditions. Furthermore, only three of the 27 isolates showed susceptibility toward the bacteriophages they were exposed to, after the experiments.

In wells with an MOI_input_ of 10, the starting concentrations were 10^7^ PFU/ml. At the end, final phage concentrations ranged between 10^6^ and 10^7.5^ PFU/ml and were therefore very similar to the phage concentrations at the start of the experiment. However, final concentrations in the experiments using Cj18 with the phages CP74-2c1 or CP132-3c were lower and ranged between 10^4^ and 10^5^ PFU/ml. The final concentrations exceeded the starting concentrations of 10^3^ PFU/ml noticeably by 10^3^–10^4.5^ PFU/ml at the end of the experiments with an MOI_input_ of 0.001. No phages could be recovered after the growth experiments of Cj18 at an MOI_input_ of 0.001 in combination with the phages CP74-2c1 and CP132-3c.

### Bacteriophage Stability Under Different pH and Temperature Conditions

**FIGURE 5:**
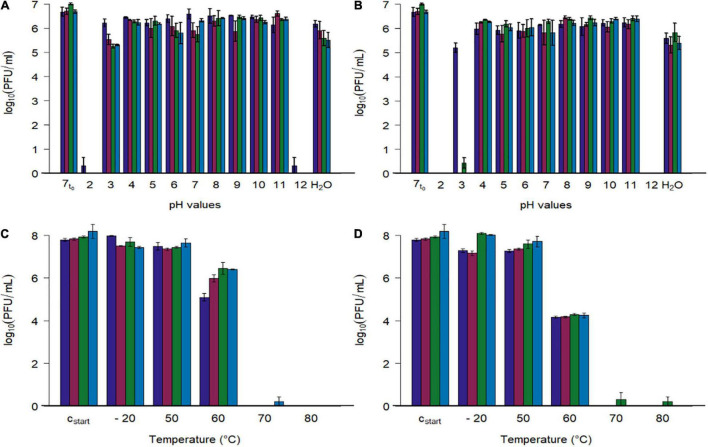
Stability tests of four phages at different pH values and different temperatures. **(A,B)** Phage concentration after exposure to different pH values (2–12) and tab water (H_2_O) at two temperatures (**A**: 22.3 ± 0.4°C, **B**: 41.7 ± 0.4). **(C,D)** Phage concentration after exposure to different temperatures (–20, 50, 60, 70, 80°C) at pH 7.4 (**C**: 15 min, **D**: 60 min). Initial concentration of SM-buffered phage solutions was 10^8^ plaque forming units/ml (c_*start*_), which were diluted tenfold with pH adjusted phosphate buffer for the first experiment (**A,B**, 7_*t*0_ = 1:10th dilution in pH 7 buffer at 0 h). Phage identity is represented as followes (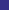 vB_CjM-LmqsCP1-4, 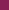 vB_CjM-LmqsCP1-5, 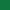 vB_CjM-LmqsCP74-2c1 and 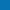 vB_CjM-LmqsCP132-3c). Columns represent the mean of the log_10_ transformed concentrations; experiments were performed in triplicate and error bars indicate the standard error of the mean.

To determine the pH stability of the investigated phages, they were diluted 10-fold in pH adjusted phosphate buffered solutions, and exposed to temperatures of 22 and 42°C for 24 h (Figures 5A,B).

All four bacteriophages lost their lytic activity when exposed to pH values below 3 or above 11 at 22 or 42°C for 24 h (Figure 5), while they remained active after being exposed to pH values ranging from 4 to 11 or tap water under similar conditions. Results show that exposing the phages to buffer solutions with a pH value of 2 at 42°C immediately decreased their activity below detection limit, while after 24 h at pH 3 and 22°C, the analyzed phages retained their lytic activity. After 24 h at pH 3 and 22°C, the mean concentrations of the phages CP1-5, CP74-2c1, and CP132-3c were significantly reduced (CP1-5: 10^5.54^ PFU/ml, *p* < 0.05; CP74-2c1: 10^5.27^ PFU/ml, *p* < 0.05; CP132-3c: 10^5.32^ PFU/ml, *p* < 0.05) in comparison to the controls (∼10^7^ PFU/ml starting concentration at pH 7), while this was not the case for CP1-4, with a final concentration of 10^6.22^ PFU/ml. However, after 24 h at pH 3 and 42°C, concentrations of CP1-5 and CP132-3c were reduced below detection limit, while CP1-4 was reduced to 10^5.21^ PFU/ml and only single plaques were observed for CP74-2c.

To examine the stability at pH 3 in detail, phage concentrations of the more susceptible phages CP1-5, CP74-2c, and CP132-3c were determined after 120 min at pH 3 and 42°C. The concentrations of the three phages were significantly reduced (CP1-5: 10^5.30^ PFU/ml, *p* < 0.05; CP74-2c1 10^3.57^ PFU/ml, *p* < 0.05; CP132-3c: 10^4.69^PFU/ml, *p* < 0.05) when compared to concentrations at the beginning of the experiment. The obtained data were used to calculate reduction rates by linear regression. CP1-5 showed the lowest reduction rate of 10^0.41^ (PFU/ml)/1 h at pH 3 and 42°C, followed by 132_3c and CP74-2c1 with respective reduction rates of 10^0.78^ (PFU/ml)/1 h and 10^1.30^ (PFU/ml)/1 h.

Subsequently, phage stability was evaluated by exposing buffered phage suspensions with titers of 10^8^ PFU/ml to –20, 50, 60, 70, and 80°C for 15 or 60 min (Figures 5C,D). Temperatures were chosen to simulate conditions during cooking or cold chain transportation. Concentrations of the four phages were not reduced during 15 min. storage at temperatures ranging from –20°C to 50°C, while their activity was completely lost at 70 and 80°C after 15 and 60 min. Concentrations of CP1-4 and CP1-5 were significantly reduced after 60 min at –20°C (CP1-4: 10^7.30^, *p* < 0.05; CP1-5: 10^7.18^, *p* < 0.05) and 50°C (CP1-4: 10^7.29^, *p* < 0.05; CP1-5 10^7.30^, *p* < 0.05) when compared to the control (∼10^8^ PFU/ml). The other phages remained stable under these conditions. After 15 min at 60°C, significant reductions in concentrations of all four phages were observed compared to the starting concentrations (CP1-4: 10^5.10^ PFU/ml, *p* < 0.05; CP1-5: 10^5.99^, *p* < 0.05; CP74-2c1: 10^6.45^ PFU/ml, *p* < 0.05; CP132-3c: 10^6.41^, *p* < 0.05), and even greater reductions occurred after 60 min.

The storage stability of the investigated phages was tested at pH 7.5 for 7 months at 4.5 ± 0.5°C and 6 weeks at 23.5 ± 0.7°C. Results of these experiments were used to determine the average reduction rates by linear regression for 1 month. At 4.5°C, the average reduction rate was calculated to range from 1.02 to 1.20 (PFU/ml)/month, while at 23.5°C, the rates increased and ranged from 1.29 to 1.82 (PFU/ml)/month for the examined phages.

### Bacteriophage DNA

For in-depth analysis, all extracted phage DNA (without an additional pre-amplification using phi29 polymerase; Kropinski et al., 2011; Crippen et al., 2019) was subjected to short-read whole-genome sequencing analysis on different Illumina Inc. devices. However, while all quality parameters of the prepared DNA and DNA sequencing libraries fulfilled the necessary requirements, no or only very low amounts of raw reads could be generated. Based on the available sequence information, no phage genome sequences could be derived.

Phage DNA was used for genome length estimations by PFGE. Estimations ranged from 144 kb for the smallest genome (CP1-4), to lengths between 148 and 152 kb for the genomes of the other three phages (CP1-5, CP74-2c1, and CP132-3c). DNA of all four phages was susceptible to cutting by the restriction enzyme HhaI (Supplementary Table 3), and real-time PCR identified them as group III phages (data not shown).

## Discussion

Nineteen *C. jejuni*-specific bacteriophages from chicken samples were isolated in this study. The host range of all isolated phages was evaluated by testing them against a panel of well-characterized *C. jejuni* and *C*. *coli* isolates, comprising current field strains and using a highly reliable spot test format. Applied methods for host range analysis vary widely among currently published studies and no validation of reproducibility for these methods is currently available (Sails et al., 1998; Hansen et al., 2007; Hwang et al., 2009; Janez et al., 2014; Sorensen et al., 2015). While some host range panels include more isolates compared to this study a representation of the current epidemiological situation in broiler production (Carrillo et al., 2005) and further information on the used strains, such as origin and typing results, were considered to be more valuable compared to a broad but less defined panel.

From the newly isolated phages, CP1-4, CP1-5, CP74-2c1, and CP132-3c were selected for further characterization. The phages were chosen based on the assumption that the origin or host range of phages might influence their performance in liquid culture. Based on their morphology, the analyzed phages could be classified as members of the *Myoviridae* in the order *Caudivirales* (Sails et al., 1998). They were further classified as group III phages as their genome sizes were similar to 138 kb, their genomes were susceptible to HhaI digestion (Sails et al., 1998; Hansen et al., 2007; Javed et al., 2014), their head sizes close to 100 nm (Sails et al., 1998), and they exclusively infected *C. jejuni* strains (Jackel et al., 2019). Subsequent real-time PCR results confirmed this identification. Results from previous studies indicated that most group III phages use capsule polysaccharide receptors to attach to their hosts (Sorensen et al., 2015; Zampara et al., 2017). Sequenced members of this group belong to the genus Fletcherviruses (Hammerl et al., 2011; Javed et al., 2014; Jackel et al., 2019; Ushanov et al., 2020). They have been used for reducing *Campylobacter* on chicken meat in different studies and proved more efficient in binding to *Campylobacter* cells at low temperatures than the flagellotropic group II phages (Atterbury et al., 2003; Goode et al., 2003; Zampara et al., 2017). While some studies did not result in significant *Campylobacter* reduction on the treated products (Orquera et al., 2012), other studies reported a significant reduction by these phages (Thung et al., 2020). Experiments on reduction of *Campylobacter* in broiler chickens by using group III phages showed significant reductions in some of the settings and varying duration of the reducing effect (Carrillo et al., 2005; Wagenaar et al., 2005; Scott et al., 2007; Kittler et al., 2013). Additionally, first trials under commercial conditions were carried out, showing the general suitability of group III phages for use in food production settings (Kittler et al., 2013).

Whole-genome sequencing of phage genomes is mandatory for final evaluation of their suitability and approval in most countries. However, in accordance with results of our study, genome determination of *Campylobacter* phages has been described to be challenging due to (i) problems regarding preparation of necessary DNA amounts and (ii) the presence of DNA modification hampering DNA processing by using enzymatic procedures. Thus, the suitability of alternative sequencing procedures (i.e., use of different DNA library preparation procedures, alternative sequencing approaches) needs to be determined. It needs to be pointed out here that phage DNA pre-amplification with Phi29 polymerase has been shown as an appropriate possibility to omit enhancing effects for DNA sequencing and manipulation for some *Campylobacter* phages (Kropinski et al., 2011; Crippen et al., 2019). Further studies need to be conducted to check whether the phage genomes of this study can be also determined by conducting this additional amplification step. However, if possible further amplification steps (PCR) should be omitted to yield a low level of nucleotide alterations in the genomes caused by additional processing (i.e., PCR amplification).

To evaluate the efficiency of the selected phages in reducing *Campylobacter*, two *C. jejuni* field strains (Cj18 and LH86), which were susceptible to all four phages, were exposed to those phages at different MOI_input_ for 26 h [similar to the experiments by Carrillo et al. (2005) or Scott et al. (2007)]. All phages were able to reduce bacterial growth at an MOI_input_ of 10 (Figure 3), resulting in lower cell densities and reduced AUC values compared to the untreated controls (Figure 4). While significant reductions in AUC values were observed in most experiments using a lower MOI_input_, the experiments using Cj18 with the phages CP74-2c1 and CP132-3c did not result in decreased AUC’s (Figure 4A). They showed a substantial decrease of the AUC in experiments using an MOI_input_ between 1 and 10, indicating lysis without replication (passive inundation), as was suggested for a similar case by El-Shibiny et al. (2009).

However, when comparing AUC values of experiments using different MOI_input_ (Figure 4), they do not indicate a dose dependence. Moreover, after ∼ 20 h, many OD_600_ values for MOI_input_ 10 were higher than those for MOI_input_ 0.001, indicating a potential negative correlation between MOI_input_ and OD_600_ values, while a positive correlation was expected as found in experiments by Rajnovic et al. (2019) for *E. coli* bacteriophages (Rajnovic et al., 2019). In 2009, El-Shibiny et al. (2009) reported similar results for *in vivo* tests with the group II phage CP220. In this study, the lowest phage dose resulted in the highest reduction in *C. jejuni* HPC5 cell numbers in chickens.

At the start of the growth experiment, phages were added at a concentration of ∼10^7^ PFU/ml. Phage titers at the end of the experiment showed similar concentrations within 10^6^–10^8^ PFU/ml, while bacteria growth was indicated by high OD_600_ value (see above). The reasons for finding high phage and bacteria numbers at the same time at an MOI_input_ of 10 are unclear, but mathematical models (Levin et al., 1977; Cairns et al., 2009; Bull et al., 2014; Loessner et al., 2020) have predicted that phages and bacteria could coexist at high numbers. Therefore, further studies are necessary to elucidate the relationship of *Campylobacter* phages and bacteria in order to improve efficacy testing and practical phage application. In experiments using the *Campylobacter* isolate LH86 and the phages CP74-2c1 and CP132-3c at an MOI_input_ below 0.1, an initial rise and subsequent decline in OD_600_ values were observed (Figures 3G,H). Similar observations were made during experiments using *E. coli* as host bacteria in other studies (Bull et al., 2014; Tolen et al., 2018; Rajnovic et al., 2019). It was assumed that except for Cj18 with CP74-2c1 and CP132-3c, the other *Campylobacter* populations could show a similar growth but this was masked by the detection limit at low OD_600_ values. Further tests are needed for a better understanding of these observations and their relevance for practical application.

In experiments combining Cj18 with CP74-2c1 or CP132-3c, significantly reduced AUC values were observed at an MOI_input_ of 10 only (Figure 4A), while an MOI_input_ of 1 or lower resulted in AUC values equal to or in one instance even significantly higher than the control (Figure 4A). Results from host range analysis indicated phage amplification, but showed reduced EOP values for the two phages on this strain. Experiments with an MOI_input_ of 10 and 0.001 resulted in reduced final phage concentrations that were 2–3 log_10_ units lower compared to the starting concentrations. These findings indicate that the loss of virions was not compensated by virion production, potentially explaining the finding that bacterial reduction only occurred if phage concentrations exceeded bacterial concentrations. In this case, bacterial reduction would have relied on initial phage concentrations and not on phage replication. Similar results of non-lytic interactions impacting bacterial growth have been reported by a previous study (Carrillo et al., 2005).

Although the number of bacterial isolates tested for phage susceptibility was limited and carful interpretation is necessary, all tested combinations of bacteria and phages resulted in resistant bacterial colonies at the end of the experiment. Interestingly, Cj18 colonies that were resistant against CP74-2c1 and CP132-3c were not resistant against CP1-4 and CP1-5. This could indicate that these phages infected bacteria by using different recognition sites. However, a higher number of tested isolates and further tests would be necessary to confirm these results.

A broad host range and a high lytic efficiency are requirements for phage application under practical conditions, while stability against adverse effects in food production settings is a prerequisite for their utilization. Application of phages in primary production exposes them to room temperature at dosing and to the chicken’s body temperature of 42 °C after ingestion. Low pH values between 3 and 5 (Mabelebele et al., 2017) are encountered for 2–3 h (Svihus and Itani, 2019) when passing through the chicken’s crop, proventriculus, and gizzard. In this regard, temperature stability at 22 and 42°C in combination with different pH levels was tested as proposed by Carrillo et al. (2005). All four phage were stable in buffered solutions with pH values ranging from pH 4 to pH 11 at 22 and 42°C, while reduced stability was observed after exposure to pH 2 and 12. Thung et al. (2020) reported similar findings for the phage Cj01. Interestingly, just one bacteriophage (CP1-4), was able to retain stability at a pH of 3 at 42°C for 24 h, while the other three phages (CP1-5, CP74-2c1, and CP132-3c) proved only stable during exposure to pH 3 for at least 2 h at 42°C. At pH 3 and 22°C, the stability was less pronounced. Based on these results, the selected phages, CP1-4, CP1-5, CP74-2c1, and CP132-3c would be able to resist environmental stresses during application in primary production settings of commercial food production. CP1-4, however, showed even higher stability than *Campylobacter* phages from previous studies.

To guarantee a sufficient shelf life for storage under practical conditions and to evaluate stability on food products at retail level, the average reduction rate of phage activity per month was calculated. At 4.5°C the average reduction rate for all four phages ranged from 1.02 to 1.20 (PFU/ml)/month, which allowed to extrapolate that in 1 year of storage an average of not more than one log_10_ unit of phage activity would be lost. Similar stabilities of *Campylobacter*-specific bacteriophages were reported by Hammerl et al. (2014). At 23.5°C, the rates increased and ranged from 1.29 to 1.82 (PFU/ml)/month. These results show a sufficient stability of the selected phages for food production settings. As the phages proved stable at temperature levels found in household refrigerators, it became important to show that heat treatment, as it would occur during food preparation, was sufficient to inactivate the bacteriophages. An exposure to 70°C for 15 min was sufficient to deactivate all four bacteriophages, while 15 min at 60°C already caused a significant reduction in phage titers.

The reductions in concentrations of CP1-4 and CP1-5 after 60 min at –20 and 50°C are unexpected (Figure 5D), as this would indicate short-term stability at –20 and 50°C. However, results presented by Figures 5A,B contradict this conclusion, as they prove a high stability of these two phages at 22 and 42°C at pH 7 for 24 h. Interestingly, the increased stability of CP1-4 against acidic pH values did not correspond to an increased temperature stability. This combination of high pH-stability and easy heat inactivation would be a favorable combination in phages used in a farm to fork approach.

## Concluding Remarks

Phage CP1-4 proved to be the most promising candidate for a wide variety of applications, as it provides a broad host range, high stability at low pH values, and heat inactivation at moderate temperatures. The phage CP1-5 originated from the same sample as CP1-4, however, its host range included five bacteria less than that of CP1-4. It appeared equally efficient in bacteria reduction based on growth experiments in liquid culture and displayed a significantly reduced pH stability, illustrating that phages with different characteristics can be isolated from the same sample.

On the other hand, the phages CP74-2c1 and CP132-3c have been rejected for commercial use based on missing efficiency at medium and lower phage doses in liquid cultures with one of the selected field strains. The obtained results illustrate the need for kinetic tests (optical density based or otherwise) on different *Campylobacter* to elucidate overall phage performance, as host range analysis with only laboratory strains does not allow for a rational phage choice. Problems during DNA extraction as well as the presence of extensive repetitive sequences on the genomes of *Camplyobacter* phages make the genetic evaluation laborious and force a search for better analysis protocols (Sorensen et al., 2021).

## Data Availability Statement

The original contributions presented in the study are included in the article/Supplementary Material, further inquiries can be directed to the corresponding author/s.

## Author Contributions

CK and SK did funding acquisition. SK and SS did study design and planning. Bacteriophage isolation was performed by GS and SK, DNA isolation was done by SS and CJ. Bacteriophage genome sequencing was done by JH, electron micrographs were taken by MR, host range determination was jointly done by EP and SS, while the rest the experiments and data analysis were performed by SS. SS and SK conceptualized and wrote the draft manuscript. SS, GS, JH, CK, EP, MR, CJ, MP, and SK helped with writing and editing the final manuscript. All authors contributed to the article and approved the submitted version.

## Conflict of Interest

The authors declare that the research was conducted in the absence of any commercial or financial relationships that could be construed as a potential conflict of interest.

## Publisher’s Note

All claims expressed in this article are solely those of the authors and do not necessarily represent those of their affiliated organizations, or those of the publisher, the editors and the reviewers. Any product that may be evaluated in this article, or claim that may be made by its manufacturer, is not guaranteed or endorsed by the publisher.
