# Supplementary figures and images for: Isolation and Characterization of Group III Campylobacter jejuni–Specific Bacteriophages From Germany and Their Suitability for Use in Food Production
Source: Front Microbiol. 2021 Dec 9;12:761223. doi: 10.3389/fmicb.2021.761223 (PMC8696038; doi:10.3389/fmicb.2021.761223)

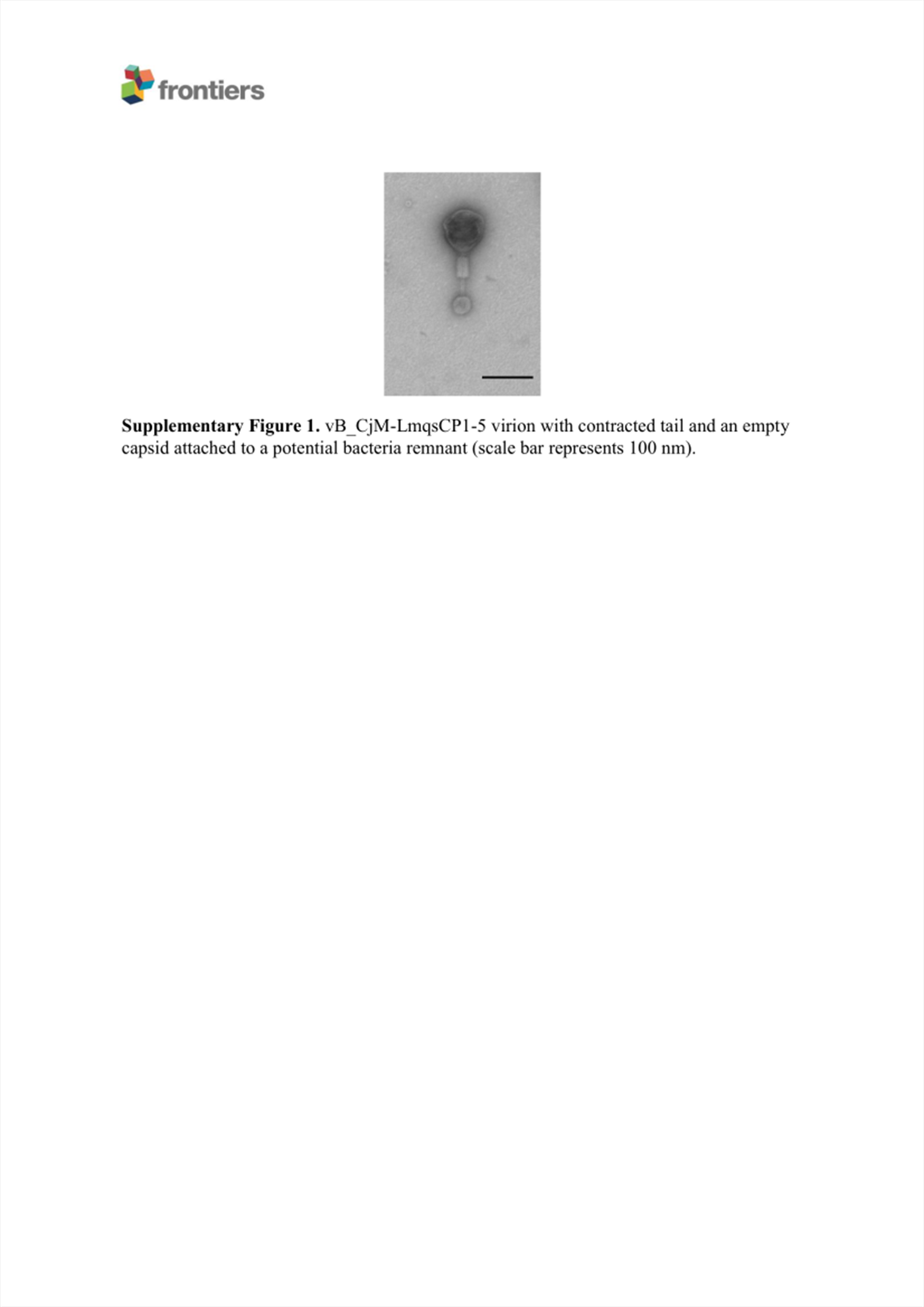

Supplement: Supplementary file 1 [file Image_1.tif]
