# Supplementary material for: Isolation and Characterization of Group III Campylobacter jejuni–Specific Bacteriophages From Germany and Their Suitability for Use in Food Production
Source: Front Microbiol. 2021 Dec 9;12:761223. doi: 10.3389/fmicb.2021.761223 (PMC8696038; doi:10.3389/fmicb.2021.761223)
Supplement: Supplementary file 2 [file Table_1.pdf]

Supplementary Table 1. *Campylobacter jejuni* and *coli* strains used in this study

Campylobacter ID	Reference	Species	Origin	flaA type	Host range
LH86	this paper	C. jejuni	chicken		α
LH73	this paper	C. jejuni	chicken		α
NCTC 12662	Health Protection Agency National Collection of Type Cultures (NCTC, Salisbury, United Kingdom) doi: 10.1128/genomeA.00969-13	C. jejuni		36	α
NCTC 11168	Health Protection Agency National Collection of Type Cultures (NCTC, Salisbury, United Kingdom) doi: 10.1038/35001088	C. jejuni	human	37	α
Cj 18	this paper	C. jejuni	chicken	117	δ
LH83	this paper	C. jejuni	chicken		ε
LH37	this paper	C. jejuni	chicken		ζ
MH3	this paper	C. jejuni	chicken		β
LH90	this paper	C. jejuni	chicken		η
Cj 16	this paper	C. jejuni	chicken		β
Cj 13	this paper	C. jejuni	chicken	975	γ
Cj 5	this paper	C. jejuni	chicken	975	θ
Cj 8	this paper	C. jejuni	chicken	975	γ
LH70	this paper	C. jejuni	chicken	49	ι
ATCC BAA-2151 (81-176)	American Type Culture Collection (ATCC, Manassas, Virginia) doi: 10.1128/JCM.43.5.2330-2338.2005	C. jejuni	human	359	κ
Cj 15	this paper	C. jejuni	chicken	22	
DSM 4689 (ATCC 33559) (NCTC 11366)	German Collection of microorganisms and cell cultures (DSMZ, Braunschweig, Germany) doi: 10.1099/00207713-45-3-592	C. coli	pig	13	
BfR-CA-09557	Bundesinstitut für Risikobewertung (BfR, Berlin, Germany) doi: 10.1186/s12864-015-2317-3	C. coli	chicken	1447	
Cc 21	this paper	*C. coli*	chicken	1596	
Cc 7	this paper	*C. coli*	chicken	16	
Cc 4	this paper	*C. coli*	chicken	1596	
Cc 2	this paper	*C. coli*	chicken	676	
LH87	this paper	*C. coli*	chicken	319	
Cc 084610	this paper	*C. coli*	chicken	70	
DSM 4688	German Collection of microorganisms and cell cultures (DSMZ, Braunschweig, Germany) doi: 10.1128/AEM.01532-10	*C. jejuni*	bovine	575	
Cj 24	this paper	*C. jejuni*	chicken	8	
Cj 10	this paper	*C. jejuni*	chicken	92	
Cj 3	this paper	*C. jejuni*	chicken	1389	
Cj 9	this paper	*C. jejuni*	chicken	975	
