# Supplementary material for: Isolation and Characterization of Group III Campylobacter jejuni–Specific Bacteriophages From Germany and Their Suitability for Use in Food Production
Source: Front Microbiol. 2021 Dec 9;12:761223. doi: 10.3389/fmicb.2021.761223 (PMC8696038; doi:10.3389/fmicb.2021.761223)
Supplement: Supplementary file 3 [file Table_2.pdf]

Supplementary Table 2. Isolated phages, sample origin and year of isolation

No.	Phage	Origin	Year
1	CP1-1 [a]	feces	2015
2	CP1-2 [a]	feces	2015
3	CP1-4 [a]	feces	2015
4	CP1-5 [a]	feces	2015
5	CP65-1	ceca	2017
6	CP73-1	skin	2017
7	CP74-2c1	skin	2017
8	CP81-1 [b]	ceca	2017
9	CP81-3 [b]	ceca	2017
10	CP113-2	skin	2017
11	CP132-3	skin	2017
12	CP134-3	ceca	2017
13	CP136-2	ceca	2017
14	CP225-3	ceca	2017
15	CP233-3	ceca	2017
16	CP235-1	skin	2017
17	CP244-3	skin	2017
18	CP264-3 [c]	ceca	2017
19	CP264-4 [c]	ceca	2017

phages originated from the same sample [a,b,c]
