# Supplementary material for: Isolation and Characterization of Group III Campylobacter jejuni–Specific Bacteriophages From Germany and Their Suitability for Use in Food Production
Source: Front Microbiol. 2021 Dec 9;12:761223. doi: 10.3389/fmicb.2021.761223 (PMC8696038; doi:10.3389/fmicb.2021.761223)
Supplement: Supplementary file 4 [file Table_3.pdf]

Supplementary Table 3. Morphologic and genomic characteristics of the examined bacteriophages. Genome size and HhaI restriction sensitivity based on PFGE gel analysis, virion dimensions based on electron micrographs and mean plaque diameters based on macrographs (± variance).

Campylobacter phage	CP1-4	CP1-5	CP74-2c1	CP132-3c
Genome length by PFGE (kb)	~ 144	~ 150	~ 152	~ 148
HhaI sensitivity	+	+	+	+
dimensions based on electron micrographs [n = 5]				
mean tail length (nm)	105.28 ± 1.86	105.34 ± 3.25	112.65 ± 4.35	106.39 ± 1.67
mean head diameter (nm)	88.49 ± 5.09	79.32 ± 4.00	88.15 ± 3.33	86.22 ± 3.43
mean head length (nm)	97.70 ± 3.57	87.73 ± 0.95	96.88 ± 3.87	100.66 ± 2.59
virus family	*Myoviridae*	*Myoviridae*	*Myoviridae*	*Myoviridae*
mean plaque diameter [24 h, 0.7% overlay, n = 30] (mm)				
	1.25 ± 0.5	1.23 ± 0.47	1.43 ± 0.4	1.34 ± 0.52
