# Supplementary material for: Isolation and Characterization of Group III Campylobacter jejuni–Specific Bacteriophages From Germany and Their Suitability for Use in Food Production
Source: Front Microbiol. 2021 Dec 9;12:761223. doi: 10.3389/fmicb.2021.761223 (PMC8696038; doi:10.3389/fmicb.2021.761223)
Supplement: Supplementary file 5 [file Table_4.pdf]

**Supplementary Table 4.** Campylobacter jejuni Cj 18 and LH86 were exposed to four different bacteriophages at different $MOI_{input}$. The resulting growth curves after 26 h were used to calculate the area under the curve (AUC). AUC was calculated by spline fitting (0 – 26 h). Phage susceptibility after 26 h incubation: (n) no visible plaque formation (with or without reduced growth of recovered *Campylobacter* isolates), (p) visible plaque formation on recovered *Campylobacter* and (bold text) indicating same phage combination as during the original experiment.

| Experimental settings | | | Area under the curve (AUC) | Phage susceptibility after experiment | | | |
|---|---|---|---|---|---|---|---|
| Bacteria strain | Phage | $MOI_{input}$ | | CP1-4 | CP1-5 | CP74-2c1 | CP132-3c |
| Cj18 | | | 1.35 | | | | |
| | CP1-4 | 10 | 0.19 | **p** | p | n | n |
| | | 1 | 0.32 | | | | |
| | | 0.1 | 0.14 | | | | |
| | | 0.01 | 0.41 | | | | |
| | | 0.001 | 0.04 | **n** | n | n | n |
| | CP1-5 | 10 | 0.20 | n | **n** | n | n |
| | | 1 | 0.20 | | | | |
| | | 0.1 | 0.25 | | | | |
| | | 0.01 | 0.19 | | | | |
| | | 0.001 | 0.24 | n | **n** | n | n |
| | CP74-2c1 | 10 | 0.23 | p | p | **n** | n |
| | | 1 | 1.54 | | | | |
| | | 0.1 | 1.55 | | | | |
| | | 0.01 | 1.94 | | | | |
| | | 0.001 | 1.54 | p | p | **n** | n |
| | CP132-3c | 10 | 0.34 | p | p | n | **n** |
| | | 1 | 1.56 | | | | |
| | | 0.1 | 1.70 | | | | |
| | | 0.01 | 1.65 | | | | |
| | | 0.001 | 1.62 | p | p | n | **n** |
| LH86 | | | 1.99 | | | | |
| | CP1-4 | 10 | 0.67 | **n** | n | n | n |
| | | 1 | 0.66 | | | | |
| | | 0.1 | 0.48 | | | | |
| | | 0.01 | 0.40 | | | | |
| | | 0.001 | 0.30 | **n** | n | n | n |
| | CP1-5 | 10 | 0.45 | n | **n** | n | n |
| | | 1 | 0.47 | | | | |
| | | 0.1 | 0.45 | | | | |
| | | 0.01 | 0.30 | | | | |
| | | 0.001 | 0.33 | p | **p** | n | n |
| | CP74-2c1 | 10 | 0.73 | p | p | **p** | n |
| | | 1 | 0.66 | | | | |
| | | 0.1 | 0.44 | | | | |
| | | 0.01 | 0.46 | | | | |
| | | 0.001 | 0.61 | n | n | **n** | n |
| | CP132-3c | 10 | 0.71 | p | p | n | **n** |
| | | 1 | 0.68 | | | | |
| | | 0.1 | 0.34 | | | | |
| | | 0.01 | 0.43 | | | | |
| | | 0.001 | 0.55 | n | n | n | **n** |
